# Mobile Phone-Mediated Interventions to Improve Adherence to Prescribed Treatment in Chronic Obstructive Pulmonary Disease: A Systematic Review

**DOI:** 10.3390/arm93020008

**Published:** 2025-04-18

**Authors:** Andrea Paleo, Catalina Carretta, Francisca Pinto, Estefanno Saltori, Joaquín González Aroca, Álvaro Puelles

**Affiliations:** School of Kinesiology, University of La Serena, La Serena 1700000, Chile; catalina.carretta@userena.cl (C.C.); francisca.pinto@userena.cl (F.P.); estefanno.saltori@userena.cl (E.S.); joaquin.gonzalez@userena.cl (J.G.A.); apuelles@userena.cl (Á.P.)

**Keywords:** Chronic Obstructive Pulmonary Disease, treatment adherence, mobile phone-mediated interventions, digital health

## Abstract

**Highlights:**

**What are the main findings?**

- The included studies were characterized by small sample sizes, short intervention durations, and methodological heterogeneity.
- The current evidence is insufficient to draw definitive conclusions about the effectiveness of mHealth-based interventions in improving treatment adherence among COPD patients.

**What is the implication of the main finding?**

- Future studies should include larger samples.

**Abstract:**

Chronic Obstructive Pulmonary Disease (COPD) is a progressive respiratory disorder that poses significant challenges in treatment adherence. This systematic review evaluates the effectiveness of mobile health (mHealth) interventions compared to conventional therapy in improving treatment adherence among COPD patients. A total of 13 randomized controlled trials and comparative cohort studies published up to July 2023 were included, analyzing interventions such as medication reminders and remote monitoring in adult COPD patients. Studies involving participants under 18 years of age or with severe comorbidities were excluded. This review identified 4688 records from MEDLINE, WEB OF SCIENCE, and SCOPUS. Of these, 13 studies met the inclusion criteria. The selection process was conducted by two independent reviewers, with discrepancies resolved by consensus with a third reviewer. The results showed that mHealth interventions improved treatment adherence in some studies, particularly in exercise and symptom monitoring; however, the evidence was inconsistent, and results varied across studies. This review concludes that mHealth interventions have the potential to improve adherence but higher-quality studies and more robust designs are needed to confirm these findings and support their clinical implementation.

## 1. Introduction

Chronic Obstructive Pulmonary Disease (COPD) is a progressive respiratory condition that represents one of the leading causes of morbidity and mortality worldwide, affecting more than three hundred million people and accounting for approximately three million deaths each year [1,2]. This disorder is characterized by persistent airflow obstruction and a chronic inflammatory response in the lungs [3,4]. The management of COPD involves a complex therapeutic regimen that includes pharmacotherapy, pulmonary rehabilitation, and lifestyle modifications, with strict adherence to these treatments being essential to prevent exacerbations and improve patients’ quality of life [5].

However, achieving optimal adherence to prescribed interventions in COPD patients remains a significant challenge. Studies suggest that up to 50% of patients fail to adhere to their treatment [6], which results in a higher risk of exacerbations, recurrent hospitalizations, and an accelerated decline in lung function [7]. Among the multiple barriers to adherence are the complexity of the treatment, patients’ negative perceptions of their disease, and logistical difficulties in accessing healthcare services [8]. In this context, mobile health (mHealth) interventions have emerged as a promising tool to address these challenges, providing medication reminders, remote monitoring, and personalized educational support through mobile devices [9,10,11]. mHealth interventions offer a unique opportunity to improve disease self-management and promote treatment adherence by providing continuous access to information and direct communication with healthcare providers [11,12]. However, despite their growing popularity, the effectiveness of these interventions in improving treatment adherence in COPD patients has yet to be fully established. The available studies present diverse, and in some cases inconsistent, results due to differences in study design, the types of interventions applied, and the methodological quality of the clinical trials [13,14,15].

Given the lack of consensus regarding the efficacy of mHealth interventions in improving adherence in COPD patients, this systematic review aims to assess the effect of mobile phone-mediated interventions to improve adherence to prescribed treatment in COPD subjects compared to conventional therapy. Specifically, it will analyze studies that investigate the use of medication reminders, remote monitoring, and other mobile applications designed to support the management of COPD.

## 2. Materials and Methods

This systematic review was conducted according to the PRISMA guidelines [16] and the protocol was registered in the International Prospective Register of Systematic Reviews (PROSPERO; ID: CRD42023455996).

### 2.1. Criteria for Considering Studies for This Review

#### 2.1.1. Types of Studies

We included randomized controlled trials (RCTs), with no restrictions on language or date of publication. Studies had to be available in full text, whether published or not.

#### 2.1.2. Types of Participants

RCTs were included if participants had a medical diagnosis of Chronic Obstructive Pulmonary Disease (COPD) confirmed by spirometry, following the criteria established by the Global Initiative for Chronic Obstructive Lung Disease (GOLD) and the American Thoracic Society (ATS).

Exclusion criteria included RCTs with populations unable to effectively use mobile health (mHealth) devices. Inability was determined by factors such as advanced age, lack of access to mobile digital devices, or significant physical or cognitive impairments that hindered the use of these technologies.

#### 2.1.3. Types of Interventions

This review included trials comparing mHealth-based educational programs with other interventions or no intervention. The mHealth interventions evaluated in this review included the following:

Internet-based learning platforms: These platforms were designed for educational purposes and delivered content through web browsers accessible on smartphones, tablets, or computers. They included interactive tools, videos, and educational modules tailored to improve disease management and treatment adherence;

Mobile applications (apps): Specific apps installed on smartphones or tablets were used to provide reminders, track symptoms, and monitor medication adherence. These apps often included features such as progress tracking, motivational messages, and real-time feedback from healthcare professionals;

SMS-based interventions: Text message (SMS) programs sent automated reminders for medication schedules, exercise routines, or symptom monitoring. Some SMS systems also allowed for two-way communication with healthcare professionals to provide additional support.

These mHealth interventions aimed to address common barriers to treatment adherence, such as lack of motivation, limited access to healthcare facilities, and insufficient patient knowledge. By leveraging mobile technologies, these interventions sought to provide accessible, personalized, and scalable solutions to improve health outcomes in individuals with chronic conditions such as COPD.

#### 2.1.4. Types of Outcomes Measures

- Primary outcome: Adherence to treatment
- Secondary outcomes: Ability to Perform, Physical Activity, Lung Function, Quality of Life, Hospitalizations

### 2.2. Search Methods for Identification of Studies

We searched the following databases for relevant trials up to July 2023:

- MEDLINE (PubMed);
- WEB OF SCIENCE;
- SCOPUS.

The search strategy was designed based on relevant medical subject heading terms (MeSH) with the following combinations:

#1 “Chronic Obstructive Pulmonary Diseases” OR “Chronic Obstructive Lung Disease” OR COAD OR COPD OR “Airflow Obstruction” OR “Chronic Airflow Obstructions” OR Smokers;

#2 Mobile OR Cellphone OR phone OR Smartphone OR mhealth OR App OR Applications OR eHealth;

#3 “Treatment Compliance” OR “Treatment Adherence” OR Adherence OR “Patient Compliance” OR “Self efficacy” OR Exacerbations OR “Physical activity” OR “Adverse events” OR “Emergency care” OR “Tobacco abstinence” OR Dyspnoea OR “Self care”;

#4. #1 AND #2 AND #3.

### 2.3. Data Management

In the data management process for this systematic review, several tools and methodologies were implemented to ensure the efficient and accurate organization of records:

Duplicate Removal:

Covidence software, in its free version, was used to identify and eliminate duplicate records. The automated process in Covidence ensures precise removal by comparing titles, authors, publication dates, and sources, minimizing human error and guaranteeing that only unique records are processed in subsequent stages;

Manual Screening:

The remaining records, after duplicate removal, were exported to Rayyan, an open-access tool that enables reviewers to tag, include, or exclude records in a blind and parallel manner, promoting transparency and minimizing bias in study selection;

Storage:

All references were organized and stored in the Zotero bibliographic manager, which allowed for maintaining a structured record of sources and facilitated the generation of citations and bibliographies in standardized formats.

These tools worked complementarily to optimize data management, ensuring an orderly and reproducible workflow. This approach guarantees that subsequent stages of the review, such as screening and data extraction, are conducted efficiently and with high accuracy.

### 2.4. Study Selection Process and Data Extraction

The study selection and data extraction process was conducted in three main stages:

1. Initial Record Selection:
  Two reviewers (C.C. and E.S.) independently and blindly assessed the records to ensure unbiased evaluation. Discrepancies were resolved by consensus, and if unresolved, a third reviewer (F.P.) mediated to reach an agreement;
2. Title and Abstract Screening:
  After duplicates were removed, reviewers screened titles and abstracts based on predefined inclusion criteria. This step efficiently reduced the number of records for full-text review;
3. Full-Text Review:
  Articles selected from the initial screening were thoroughly reviewed to determine eligibility, focusing on study methodology, participant characteristics, interventions, and outcomes.

Discrepancies identified during the screening or review stages were resolved through discussion between the primary reviewers, with the third reviewer providing additional input when required. This process ensured methodological rigor and minimized bias.

### 2.5. Data Extraction

The data extraction process was designed to ensure methodological rigor, consistency, and accuracy in collecting relevant information from the included studies. The procedure involved the following key steps:

1. Independent data extraction: Two reviewers (C.C. and E.S.) independently extracted data using a standardized extraction form. This form was developed based on the study objectives and predefined inclusion criteria, ensuring consistent collection of relevant information across all studies;
2. Discrepancy resolution: Discrepancies identified during the independent extraction process were discussed and resolved through consensus between the two reviewers (C.C. and E.S.). For unresolved discrepancies, a third reviewer (F.P.) was consulted to provide an impartial perspective. This collaborative approach ensured that all extracted data were accurate and aligned with the study objectives;
3. Validation of extracted data: to maintain data quality, the final dataset was reviewed and verified by all three reviewers (C.C., E.S., and F.P.) to ensure completeness and accuracy;
4. Tools and documentation: all extracted data were systematically documented in the standardized form and stored in a central repository, ensuring traceability and facilitating the subsequent analysis.

### 2.6. Assessment of Risk of Bias in Included Studies

The risk-of-bias assessment for the included studies was conducted using a rigorous and standardized approach with the Cochrane risk-of-bias tool for randomized trials (RoB 2) [17]. This process included the following key steps to ensure accuracy and consistency:

1. Independent assessment by reviewers: Two reviewers (C.C. and E.S.) independently evaluated the risk of bias for each study. This independent evaluation aimed to reduce subjectivity and prevent mutual influence between reviewers. The RoB 2 tool was systematically applied to assess bias, following a predefined set of criteria that ensured uniformity in judgments across all studies;
2. Risk-of-Bias Assessment Domains:
  The following domains, as outlined in the Cochrane Handbook, were considered during the evaluation:
  ○ Evaluation of the adequacy of random sequence generation and allocation concealment;
  ○ Analysis of whether deviations from the assigned interventions affected the outcomes;
  ○ Examination of the extent and reasons for missing outcome data and their impact on the study’s results;
  ○ Determination of whether the measurement of outcomes was influenced by knowledge of the intervention;
  ○ Identification of selective reporting of outcomes that could distort the study’s findings;
3. Discrepancy Resolution:
  ○ Discrepancies between the two reviewers were addressed through discussions and consensus;
  ○ If no agreement was reached, a third reviewer (F.P.) was consulted to mediate and finalize the evaluation. This collaborative process ensured that all judgments were well-founded and consistent;
4. Peer Review of Bias Assessments:
  ○ After the initial evaluations, the results were reviewed by the third author (F.P.) and discussed with the two primary reviewers. This step added an additional layer of oversight to confirm the reliability of the assessments.

## 3. Results

**Figure 1:**
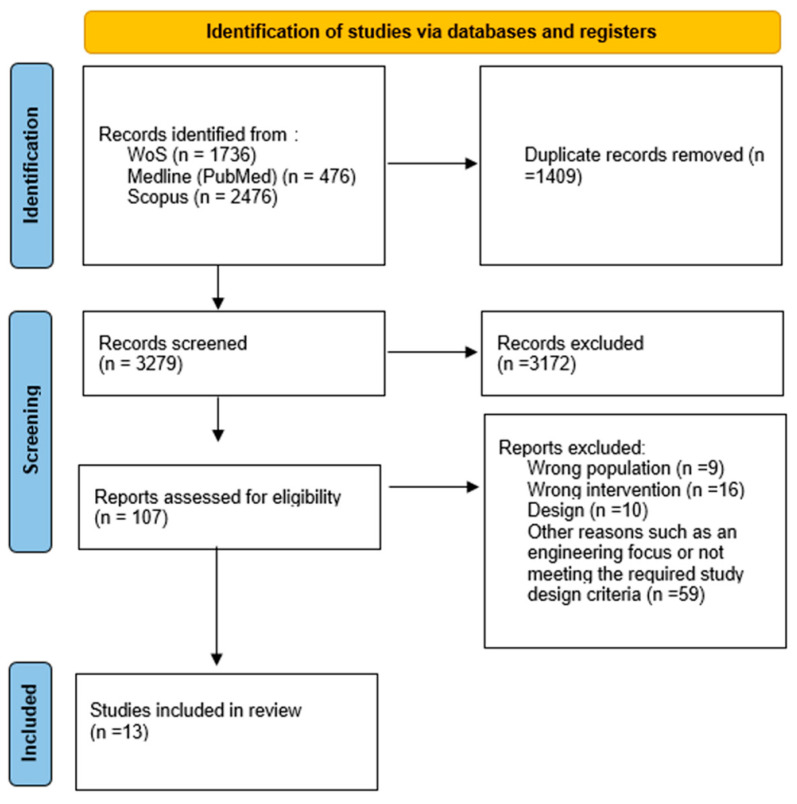
Flow diagram of the literature search and selection process.

The literature search resulted in 4688 records. After duplicate removal, 3279 records. During title and abstract screening, we excluded 3172 clearly irrelevant records. We proceeded to retrieve the full-text reports for 107 records. Of these, we excluded 94 studies for reasons summarized in Figure 1.

### 3.1. Characteristics of the Included Studies

The characteristics of the included studies are summarized in Table 1. A total of 1167 participants were involved, of which 715 were men (61.26%). The average age was 67.12 years. All participants were diagnosed with COPD using spirometry, with the GOLD criteria being the most commonly used, followed by ATS. The average stage of the disease was between II and III.

Regarding the different interventions conducted in the included studies, three major categories were identified: follow-up calls, messaging services (either as reminders or to promote treatment adherence), and activity logging. As for the comparators, the most frequently used was usual care, followed by a comparison with groups that received no intervention (wait-and-see approach).

Finally, in terms of the outcomes recorded across the different studies, the most prevalent was treatment adherence, followed by the ability to perform exercise or physical activity, and behavioral changes associated with the disease [18,19,20,21,22,23,24,25,26,27,28,29].

**Table 1:** General characteristics of the included studies.

Study	Participant Characteristics Health Condition	Participant Characteristics Intervention Group	Participant Characteristics Control Group	Objectives	Intervention	Outcomes
Galdiz et al. [18] (Spain, 2020)	Moderate-to-severe COPD (BODE index 3–7).	Number of participants: 46 Mean age: 62.3 years Male/female ratio: 65.2% male, 34.8% female	Number of participants: 48 Mean age: 63 years Male/female ratio: 68.8% male, 31.2% female	Evaluate pulmonary rehabilitation using a home kit.	Intervention group: pulmonary rehabilitation with a mobile app, pulse oximeter, weights, and stationary bike at home. Control group: recommendation for regular exercise, general educational materials, and periodic clinical follow-ups.	Primary outcome: exercise tolerance and changes in SF-36 quality of life.
Ko Wai-San et al. [26] (Hong Kong, 2020)	COPD ≥ 40 years, hospitalized for AECOPD.	Number of participants: 68 Mean age: 76 years Male/female ratio: 99% male, 1% female	Number of participants: 68 Mean age: 74 years Male/female ratio: 95% male, 5% female	Reduce hospital readmissions due to AECOPD.	Intervention group: biweekly mobile calls combined with standard care. Control group: intervention limited to usual care, with no specific strategies to reinforce physical activity or provide additional support such as telephone follow-ups.	Primary outcome: hospital readmissions due to AECOPD.
Wang et al. [19] (China, 2021)	COPD patients aged 40–80 years with access to smartphones.	Number of participants: 39 Mean age: 63.2 years Male/female ratio: 66.7% male, 33.3% female	Number of participants: 39 Mean age: 64.4 years Male/female ratio: 74.4% male, 25.6% female	Analyze the impact of mHealth on self-management.	Intervention group: mHealth program with educational modules and expert support. Control group: basic health education and standard medical care.	Primary outcome: health-related quality of life.
Çevirme et al. [20] (Turkey, 2020)	COPD Stage II for at least 6 months	Number of participants: 20 Mean age: 66 years Male/female ratio: 55% male, 45% female	Number of participants: 20 Mean age: 61 years Male/female ratio: 65% male, 35% female	Evaluate mobile education for self-management.	Intervention group: mobile education for self-care and disease management. Control group: standard follow-up at the hospital, usual medical treatment.	Primary outcome: changes in dyspnea and self-care.
Sink et al. [27] (EE.UU., 2018)	COPD diagnosed within the last 24 months, capable of receiving text/voice messages.	Number of participants: 83 Mean age: 59.89 years Male/female ratio: 29 males/54 females	Number of participants: 85 Mean age: 61.9 years Male/female ratio: 32 males/53 females	Examine the impact of daily messages on symptom management.	Intervention group: automated daily messages for symptom monitoring. Control group: received automated daily messages monitoring respiratory status but no alerts were sent to healthcare providers when symptoms worsened.	Primary outcome: time to hospitalization.
Boer et al. [30] (Nijmegen, 2018)	COPD with ≥2 exacerbations in the last 12 months.	Number of participants: 43 Mean age: 69 years Male/female ratio: 58% male, 42% female	Number of participants: 44 Mean age: 65 years Male/female ratio: 66% male, 34% female	Evaluate the effect of mHealth tools alongside pulmonary rehabilitation.	Intervention group: daily use of mHealth tools during pulmonary rehabilitation. Control group: use of a paper-based action plan to manage COPD exacerbations.	Primary outcome: number of exacerbations.
Vorrink et al. [21] (The Netherlands, 2016)	COPD (GOLD stage 2–3), completed pulmonary rehabilitation within the last 6 months.	Number of participants: 102 Mean age: 62 years Male/female ratio: 50% male, 50% female	Number of participants: 81 Mean age: 63 years Male/female ratio: approximately 50% male, 50% female	Analyze the impact of mHealth on physical activity.	Intervention group: mHealth app with physiotherapist support. Control group: usual care provided by physicians.	Primary outcome: physical activity levels.
Walters et al. [22] (Australia, 2013)	COPD patients > 45 years with confirmed diagnosis by spirometry.	Number of participants: 90 Mean age: 68 years Male/female ratio: 51% male, 49% female	Number of participants: 92 Mean age: 63 years Male/female ratio: 54% male, 46% female	Evaluate the effectiveness of telephone-based health mentoring in COPD patients.	Intervention group: regular health mentoring calls. Control group: usual care by general practitioners and regular follow-up calls from research nurses.	Primary outcome: changes in SF-36 quality of life.
Tabak et al. [23] (The Netherlands, 2013)	COPD without exacerbation in the last 4 weeks	Number of participants: 18 Mean age: 65 years Male/female ratio: 8 males/6 females	Number of participants: 18 Mean age: 65 years Male/female ratio: 8 males/6 females	Evaluate the impact of an mHealth app.	Intervention group: app with activity tracking and symptom monitoring. Control group: local physiotherapy and standard medication.	Primary outcome: activity levels measured with a pedometer.
Chau et al. [29] (Hong Kong, 2013)	COPD patients ≥ 60 years with moderate-to-severe disease.	Number of participants: 22 Mean age: 73 years Male/female ratio: 95.5% male, 4.5% female	Number of participants: 18 Mean age: 72 years Male/female ratio: 100% male	Evaluate the use of mobile monitoring.	Intervention group: mobile monitoring three times a day (oxygen saturation, pulse, respiration). Control group: usual care.	Primary outcome: satisfaction and health-related quality of life.
Halpin et al. [24] (The United Kingdom, 2013)	COPD patients ≥ 40 years with confirmed diagnosis by spirometry.	Number of participants: 40 Mean age: 68 years Male/female ratio: approximately 74% male, 26% female	Number of participants: 39 Mean age: 70 years Male/female ratio: approximately 73% male, 27% female	Evaluate a smartphone-based intervention.	Intervention group: BlackBerry smartphones for symptom monitoring and data collection. Control group: usual care.	Primary outcome: number of exacerbations.
N. Huong et al. [25] (EE.UU., 2009)	Stable COPD with moderate-to-severe disease as defined by GOLD.	Number of participants: 9 Mean age: 72 years Male/female ratio: 33% male, 67% female	Number of participants: 8 Mean age: 64 years Male/female ratio: 37% male, 67% female	Evaluate the usability of a mobile intervention and its impact on physical performance.	Intervention group: daily symptom reports and exercise monitoring using a mobile device. Control group: physical activity monitoring, individualized exercise plan, training on pedometer use, self-monitoring training, and encouragement of self-management.	Primary outcome: usability of the mobile intervention and changes in physical performance.
W.T. Liu et al. [28] (Taiwan, 2008)	COPD patients meeting GOLD criteria, aged ≥ 40 years.	Number of participants: 24 Mean age: 71 years Male/female ratio: 100% male	Number of participants: 24 Mean age: 72 years Male/female ratio: 100% male	Evaluate guided walking using a musical tempo.	Intervention group: guided walking with a musical tempo using a mobile app. Control group: a brochure and DVD containing a home exercise program, with verbal recommendations for daily unsupervised walking at home and no additional technological support or monitoring.	Primary outcome: hospital readmissions, quality of life, and physical activity.

### 3.2. Risk of Bias of Included Studies

The methodological quality of the reviewed studies showed variability across the different domains evaluated using the RoB 2 tool. Of the 13 included studies, 11 presented a moderate risk of bias. In Domain 1 (D1)—which assesses the generation and concealment of the allocation sequence—three studies [20,23,27] did not provide sufficient information to confirm that participant allocation was random and appropriate, potentially influencing the results. In Domain 2 (D2)—which examines the blinding of participants and personnel—six studies [19,21,25,28,29,30] showed deficiencies, either due to the absence of effective blinding or insufficient description of the methods used. This may have introduced performance bias, particularly in interventions where participant behavior played a key role. In Domain 3 (D3), related to missing data, four studies [23,25,27,30] reported data loss rates exceeding 15%, compromising the reliability of the results. In Domain 4 (D4)—focused on the measurement of outcomes—eight studies [18,19,23,24,26,28,29,30] exhibited issues due to the lack of blinding of outcome assessors or the use of measurement tools that were not adequately validated, which could have biased the evaluations. Finally, in Domain 5 (D5)—related to the selection of reported outcomes—four studies [20,22,28,29] lacked clear protocols, making it difficult to compare the reported outcomes with those initially planned.

**Figure 2:**
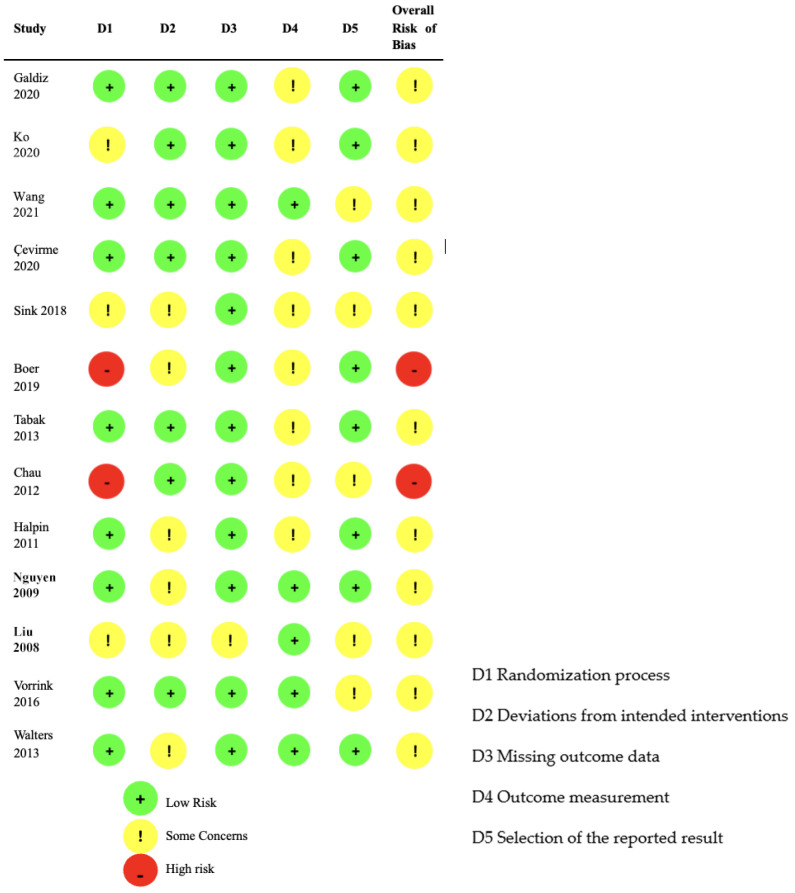
The Cochrane risk-of-bias tool for randomized trials (RoB 2) [18,19,20,21,22,23,24,25,26,27,28,29,30].

Figure 2 presents the individual risk-of-bias assessments for the included studies. Two studies were assessed as having a high risk of bias, while the remaining studies were considered to have “some concerns” according to the Cochrane tool.

### 3.3. Outcomes

#### 3.3.1. Treatment Adherence

Two studies reported results related to treatment adherence:

The first study [18] used the number of attended follow-up appointments as an indicator of adherence. Twelve patients were considered non-adherent (95% confidence interval [CI]: 8–16), resulting in a compliance rate of 60% (*p* = 0.045);

The second study [25] utilized a platform to record symptoms and exercise data, finding that the intervention group (IG) showed higher adherence (87%, 95% CI: 83–91) compared to the control group (CG) (66%, 95% CI: 61–71) over six months (*p* < 0.01).

#### 3.3.2. Ability to Perform Physical Activity

Four studies evaluated physical capacity related to treatment.

Two studies [21,23] measured the number of daily steps using pedometers:

○ In one study, both groups showed a decrease in steps over time (mean reduction: −320 steps in the CG and −280 steps in the IG; *p* = 0.12);
○ In the other study, the IG showed a non-significant increase in daily steps during the first weeks (+340 steps in week 1, 95% CI: −50 to +730; *p* = 0.09).

A third study [28] used the 6-minute walk test (6 MWT). The CG slightly increased the distance covered (baseline mean: 1200 m; at six months: 1268 m, 95% CI: 1250–1285), while the IG decreased the distance (baseline mean: 1240 m; at six months: 1194 m, 95% CI: 1170–1218; *p* = 0.02).

Another study [25] used the Incremental Shuttle Walk Test (ISWT), which showed a significant increase in the distance covered by the IG (baseline mean: 255.8 m; at eight weeks: 307.1 m, 95% CI: 298–316; *p* < 0.001).

#### 3.3.3. Lung Function

Four studies evaluated lung function.

Two studies measured forced expiratory volume in one second (FEV1) and forced vital capacity (FVC):

○ One study [26] found no significant differences in FEV1 (*p* = 0.18) or FVC (*p* = 0.22);
○ The other study [20] reported significant improvements in the IG (z = 3.103; *p* = 0.002), while differences in the CG were insignificant (z = 0.422; *p* = 0.67).

The other two studies [27,29] evaluated FEV1% and FEV1/FVC, finding no significant differences before and after the intervention in either group (*p* = 0.21).

#### 3.3.4. Quality of Life

Eight studies evaluated quality of life using questionnaires (CRQ, CCQ, SGRQ, and SF-12).

Five studies observed improvements in the IG compared to the CG:

○ In one study [18], the CRQ showed improvements in all dimensions in the IG (overall mean: +12.4 points, 95% CI: +10.2 to +14.6; *p* < 0.001);
○ Another study [22] using the SGRQ found a decrease in the CG in the short- and long-term (*p* = 0.03) but not in the IG (*p* = 0.12).

Two studies [23] found no significant differences between groups (*p* = 0.45).

One study [29] reported a greater decline in quality of life in the CG compared to the IG (SGRQ: +7.8 points in the CG vs. +2.3 points in the IG; *p* = 0.04).

#### 3.3.5. Hospitalizations

Five studies analyzed the number of hospitalizations.

Three studies [26,27,29] reported fewer hospitalizations in the IG:

○ In one study [26], the readmission rate was 13.7% in the IG (95% CI: 10–17) and 29.1% in the CG (95% CI: 24–34; *p* = 0.005).

Two studies [24,28] found more hospitalizations in the IG:

○ In one study [24], there were seven hospitalizations in the IG compared to three in the CG (*p* = 0.02).

## 4. Discussion

This systematic review evaluated the impact of mHealth-based interventions on improving treatment adherence in patients with COPD. Results from two studies [18,25] demonstrated direct and significant improvements in adherence. In the first study [18], the intervention group achieved 60% adherence to planned exercise days and 92.4% adherence to scheduled appointments compared to 84.4% in the control group. In the second study [25], participants in the intervention group showed 87% adherence to recording exercise- and symptom-related data compared to 66% in the control group. Furthermore, a correlation was observed between time spent on moderate-to-high-intensity physical activities and the frequency of symptom recording, reinforcing the positive role of mHealth tools in treatment engagement. In the remaining 11 reviewed studies [18,19,20,21,22,23,24,25,26,27,28,29], adherence was indirectly assessed, such as increased physical activity time or consistent use of mobile applications. For instance, in one study [26], a significant increase in intense physical activity time was reported in the intervention group, suggesting better adherence to therapeutic recommendations.

The discussion of these findings should not be limited to logistical and economic barriers hindering the implementation of mHealth. Other factors also influence adherence, such as advanced age, identified in several studies as a limiting factor due to difficulties in using technological devices [19,25,28]. Comorbidities, including diabetes and hypertension, complicated treatment management due to the cumulative burden of diseases [22,26]. Additionally, social isolation, especially in patients without support networks, limited the regular use of mHealth tools [27]. Another critical barrier was the lack of technological skills, reported in at least three studies [23,25,29], highlighting the need for constant technical support to ensure proper use of digital platforms.

The methodological quality of the reviewed studies showed variability across different domains assessed using the RoB 2 tool. Of the 13 included studies, 11 had a moderate risk of bias. In Domain 1 (D1), related to random sequence generation and allocation concealment, three studies [20,23,27] provided insufficient information to ensure proper randomization, potentially affecting the results. In Domain 2 (D2), evaluating the blinding of participants and personnel, six studies [19,21,25,28,29,30] had deficiencies either due to ineffective blinding or inadequately described methods, potentially introducing performance bias, particularly in behavior-dependent interventions. In Domain 3 (D3), related to missing outcome data, four studies [23,25,27,30] reported data loss rates exceeding 15%, affecting result reliability. In Domain 4 (D4), assessing outcome measurement, eight studies [18,19,23,24,26,28,29,30] faced issues due to unblinded evaluators or unvalidated measurement tools. Finally, in Domain 5 (D5), related to selective reporting, four studies [20,22,28,29] lacked clear protocols, making it challenging to compare reported results with planned outcomes. This study’s review process is detailed in the Section 2.

The findings of this review are consistent with existing evidence supporting mHealth use to improve treatment adherence in various chronic conditions. However, heterogeneity in study designs and populations limits the generalizability of the results. Despite these challenges, mHealth interventions hold significant potential to transform COPD management, especially by offering accessible solutions for patients facing geographic or economic barriers. Nonetheless, it is crucial to address the identified limitations, including technology-related barriers, sociodemographic characteristics, and contextual factors.

In conclusion, mHealth tools represent a promising opportunity to improve treatment adherence in patients with COPD. Future studies should focus on more diverse populations, address technological gaps, and improve methodological designs to validate their impact in different clinical and social contexts. Additionally, strengthening systematic review processes is necessary to ensure the quality and accuracy of results, establishing a more robust foundation for the effective implementation of these interventions in clinical and community practice.

## 5. Conclusions

The current evidence does not allow for definitive conclusions about the effectiveness of mHealth-based interventions in improving treatment adherence among COPD patients. This uncertainty arises from limitations such as small sample sizes, short intervention durations, and methodological heterogeneity across the reviewed studies. While statistically significant differences compared to conventional care are not yet evident, mHealth interventions demonstrate the potential to reduce costs, optimize resources, and enhance healthcare access for patients facing logistical barriers to regular health center visits. These tools may play a crucial role in healthcare delivery, particularly in resource-limited settings or regions with restricted access to services. However, these conclusions remain preliminary and are subject to change as more robust evidence emerges. To address the gaps in evidence, future research should focus on conducting rigorously designed randomized controlled trials (RCTs) with large sample sizes to ensure sufficient statistical power. These studies should implement robust randomization techniques, allocation concealment, and blinded outcome assessments to minimize bias. Additionally, extended follow-up periods, exceeding six months, are necessary to evaluate both immediate and long-term effects of mHealth interventions. Future research should also explore the effectiveness of different mHealth modalities across subgroups of COPD patients, considering factors such as disease severity, comorbidities, and patient preferences. Economic analyses assessing cost-effectiveness and sustainability are equally essential to determine the feasibility of integrating mHealth into routine clinical practice. As high-quality evidence accumulates, precise recommendations for incorporating mHealth technologies into COPD management can be developed, ultimately optimizing patient outcomes and healthcare efficiency.

## Data Availability

Not applicable.
